# RRAP: RPKM Recruitment Analysis Pipeline

**DOI:** 10.1128/mra.00644-22

**Published:** 2022-08-22

**Authors:** Conner Y. Kojima, Eric W. Getz, J. Cameron Thrash

**Affiliations:** a Department of Biological Sciences, University of Southern California, Los Angeles, California, USA; Indiana University, Bloomington

## Abstract

A common method for quantifying microbial abundances *in situ* is through metagenomic read recruitment to genomes and normalizing read counts as reads per kilobase (of genome) per million (bases of recruited sequences) (RPKM). We created RRAP (RPKM Recruitment Analysis Pipeline), a wrapper that automates this process using Bowtie2 and SAMtools.

## ANNOUNCEMENT

Quantifying the relative abundance of microorganisms in a sample is a critical component of microbial ecology research. Whole-community metagenomic sequencing can be used to calculate relative abundance after recruiting reads to genomes generated from isolates, metagenomes, or single cells (1–4). Since genomes will have different sizes and each sample will have different numbers of reads, normalizing for these two variables can be accomplished with the RPKM (reads per kilobase [of genome] per million [bases of recruited sequences]) method, which was originally developed to quantify relative transcript abundance (5).

To automate the process of read recruitment and RPKM normalization for use in recruiting hundreds or thousands of samples to similarly large numbers of genomes, we developed RRAP (RPKM Recruitment Analysis Pipeline). RRAP is a wrapper for other established tools that takes paired-end metagenomic sequences and reference genome sequences as the input and generates both read alignment data and RPKM values. The pipeline streamlines the read recruitment process by automatically handling the preprocessing steps of merging contigs, concatenating reference genomes, and indexing reference sequences. RRAP installs the most recent versions of Bowtie2 and SAMtools that are compatible with the other dependencies (6, 7). After performing read recruitment with Bowtie2, the pipeline sorts and indexes sequence alignment data before counting the numbers of mapped and unmapped metagenomic reads per reference sequence with SAMtools. From the output, RRAP calculates both unadjusted and log_10_-adjusted RPKM values for each reference genome in each metagenomic sample.

Other bioinformatics tools are similar to RRAP but serve different purposes. The Enveomics Collection is a compilation of scripts that analyze metagenomes (8). The scripts BlastTab.catbj.pl and BlastTab.recplot2.R in particular use BLAST results to generate a recruitment plot for visualization purposes. The script anir.rb estimates the average nucleotide identity of reads against a genome using existing alignment data. Anvi’o also provides a metagenomics workflow that assembles reads and maps them to contigs, but this is a much more comprehensive software package than RRAP and serves numerous purposes (9, 10). There are other existing pipelines that perform read recruitment but do not calculate RPKM values. Sunbeam and ngs_backbone are two examples that recruit reads with bwa instead of Bowtie2 to produce alignment data but do not calculate RPKM values (11–13). RRAP is therefore a unique, lightweight, and standalone pipeline for both recruitment and RPKM calculation.

### Data availability.

The code, detailed instructions for use, and sample data files to install and test run RRAP are available on GitHub (https://github.com/thrash-lab/rrap). Because the pipeline has dependencies, we recommend installation through the Conda package manager (14). Upon installation, RRAP can be accessed from the command line with a single command. Sample metagenomes and reference genomes to allow quick testing of read recruitment and RPKM calculations were obtained from previous studies (15–19).
